# Optical properties of nanocrystal films: blue shifted transitions as signature of strong coupling†

**DOI:** 10.1039/c9na00647h

**Published:** 2019-11-25

**Authors:** Erik S. Skibinsky-Gitlin, Salvador Rodríguez-Bolívar, Marco Califano, Francisco M. Gómez-Campos

**Affiliations:** Departamento de Electrónica y Tecnología de Computadores, Facultad de Ciencias, Universidad de Granada 18071 Granada Spain; CITIC-UGR C/Periodista Rafael Gómez Montero, n 2 Granada Spain; Pollard Institute, School of Electronic and Electrical Engineering, Bragg Centre for Materials Research, University of Leeds Leeds LS2 9JT UK m.califano@leeds.ac.uk

## Abstract

We present a theoretical study at the atomistic level of the optical properties of semiconductor nanocrystal films. We investigate the dependence of the absorption coefficient on size, inter-dot separation, surface stoichiometry and morphology, temperature, position of the Fermi level and light polarization. Our results show that, counter-intuitively, huge *blue* shifts are expected in some intra-band transitions for strongly coupled arrays, in contrast with the predicted and observed *red* shift of the band gap absorption in such systems. Furthermore, we find that the energies of such transitions can be tuned within a range of several hundreds of meV, just by engineering the inter-dot separation in the film through the choice of appropriately sized capping ligands. Finally we discuss the application of this effect to nanocrystal-based intermediate-band solar cells.

## Abstract 2

Large blue shifts in the intra-conduction-band transitions and the red shift of the band gap absorption are both the manifestation of strong interdot coupling in the film.

## Graphical abstract

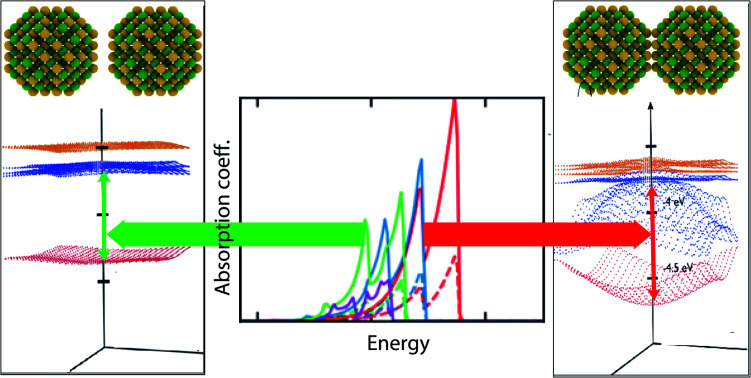

## 1. Introduction

Semiconductor nanocrystals (NCs) are widely employed as building blocks for a variety of technological applications,^1^ owing to their inexpensive synthesis and size-tunable optical properties. In most of these applications, however, the NCs are assembled in 2D or 3D arrays, *i.e.*, films or solids, whose properties exhibit both single-dot-like features, such as a well defined size-dependent absorption onset, and array behaviour, such as the formation of minibands^2,3^ and the emergence of bulk-like transport.^4^ The latter properties are strongly influenced by the distance between the NCs in such assemblies.^5–8^ We recently showed^3^ that strong coupling is possible for experimentally achievable inter-dot separations, and can lead to the formation of wide minibands, and yield high electron mobilities,^8^ depending on the NC size and material composition. One of the typical signatures attributed to strong coupling is the red shift in the absorption edge observed in the transition from dilute solutions to close-packed arrays.^4,9–12^ This feature is interpreted in terms of electron and hole wave function delocalisation over more than one dot (*i.e.*, “extended states”), leading to a reduction in carrier's confinement (an effect similar to an increase in dot size), hence to a reduction in the band gap energy. If this were the case, in analogy with the increased density of states (hence the reduced inter-level separation) exhibited by a larger dot, such delocalisation should, in principle, also produce a red shift in all intra-band transitions.

We showed in the past^3^ that both stoichiometry and surface morphology of the single dots have a strong influence on the film's miniband structure. However, how these features translate into measurable differences in the optical properties remains to be investigated.

Another interesting question concerns the effect of the Fermi level's position on the optical properties of a NC film, considering that in some situations the samples may be doped, either optically (photodoping) or electrochemically.

In order to address these issues and to investigate the direction and origin of the energy shift(s) of the different intra-conduction-band transitions, here we carry out a comprehensive theoretical study of the optical properties of nanocrystal films, using LDA-quality wave functions for the isolated dot (obtained within the atomistic semiempirical pseudopotential method^13^), as a basis set for a tight-binding calculation of the film's band structure and optical spectra. We investigate the dependence of the latter on (i) size, (ii) inter-dot separation, (iii) surface stoichiometry and (iv) morphology, (v) temperature, (vi) Fermi level's position, and (vii) light polarization. We find that the key properties determining the shift in any transition energy are specifically the width and shape of the minibands involved (which can differ in films made of NCs of the same size and material, but different stoichiometries), rather than simply the degree of deconfinement of the carriers in general: indeed such shifts can be either to the red or to the blue, depending on the relative positions (with respect to each other) of the miniband minima in the Brillouin zone, with larger shifts occurring, in decreasing order of importance, (a) for smaller dots, (b) for shorter inter-dot separations, (c) in films made of dots with anion-rich surfaces, and (d) for structured surface morphologies that allow some degree of dot interlocking. These effects are more clearly seen when the Fermi level is situated at or below the CBM-derived miniband minimum and at low temperatures.

We also show that, in the strong coupling regime, the film's optical spectrum can be very different from that of the isolated dot and therefore cannot be simply deduced from single-dot spectroscopy. Finally, based on our results we propose a new scheme for the realisation of NC-based intermediate band solar cells.

## 2 Theoretical method

**Fig. 1:**
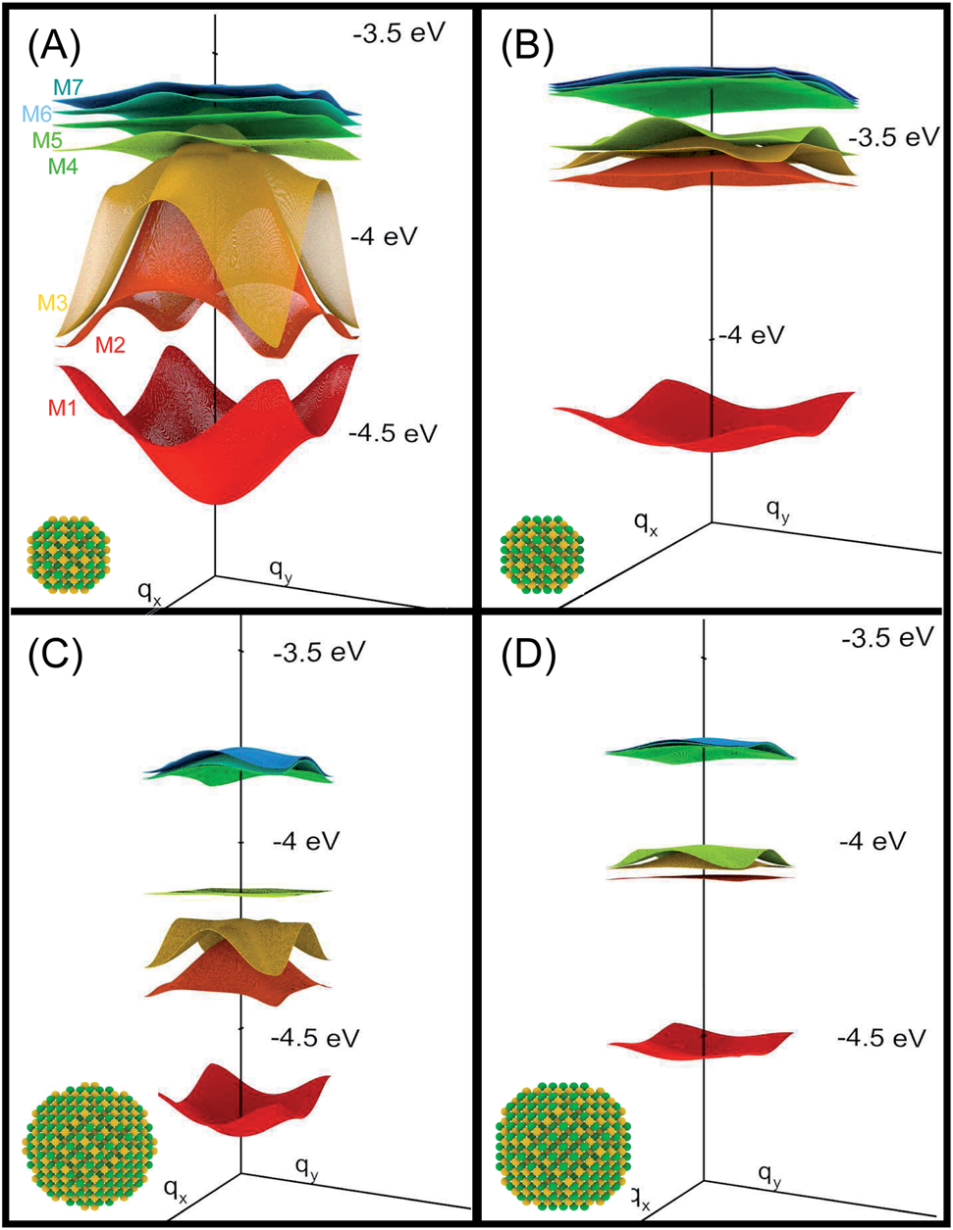
Miniband structure for the lowest conduction band states in structures A–D (type A, B and D NCs are separated by one bond length, whereas type C dots are 0.25 bond lengths apart, as in this structure the dots are interlocked through their surface As atoms^3^). The energies are referred to the vacuum level. The insets show the atomistic structure of the different systems, where yellow and green spheres represent As and In atoms, respectively.

The electronic states of the periodic system (Fig. 1) are obtained within the tight-binding approach, by expanding the superlattice wave functions in a basis of single quantum dot conduction band eigenstates obtained within the atomistic semiempirical pseudopotential framework^3^ (for further details please see ESI†).

The absorption coefficient is calculated within the electric dipole approximation using Fermi's Golden Rule, as1
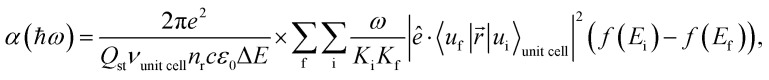
where *e* is the electron charge, *Q*_st_ is the number of vectors of the reciprocal space for which the Schrödinger equation is solved (a 501 × 501 grid discretization is used here to sample the Brillouin zone), *ν*_unit cell_ is the volume of the superlattice unit cell, *n*_r_ is the refractive index of the material (for simplicity here we use *n*_r_ = 1), *c* is the speed of light in vacuum, *ε*_0_ is the vacuum dielectric constant, Δ*E* is the interval width within which energy is assumed to be conserved, (*i.e.*, the Dirac's delta function is approximated as a box), *ω* is the angular frequency of the photon involved in the absorption process, 
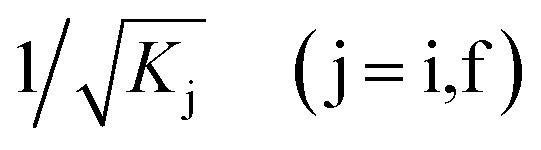
 are normalization constants, *ê* is the potential vector polarization, *u*_f_ and *u*_i_ are the Bloch functions of the superlattice wave function for the final (f) and initial (i) states, and *f*(*E*) is Fermi–Dirac's statistics.

## 3 Results

We consider InAs nanocrystal-based 2D square superlattices^14^ made using 4 different structures as building blocks: (A) cation-centered and (B) anion-centred spherical dots with *R* = 1.2 nm; (C) “rough-faceted” and (D) “smooth-faceted” spherical dots with *R* = 2.0 nm (see insets of Fig. 1). Type-B NCs are obtained from type-A ones by interchanging cations and anions, resulting in cation-rich surfaces, in contrast with the anion-rich surfaces in type-A dots. This allows us to investigate the effect of different surface stoichiometries on the absorption properties of dots of the same size. Type-D NCs are equivalent to type-B dots in that they have a cation-rich surface, only with a larger size. This allows us to isolate size effects. Type-C are similar to type-D, but have two extra As atoms on 6 of their facets, and allow us to study the effect of different surface morphologies (*i.e.*, of some degree of surface roughness that may exist in experimental samples) on otherwise same-sized dots (at the closest separation, in type C NCs the two As atoms on the surface of one dot interlock with the atoms on the surface of the other, like two LEGO bricks^3^).

### 3.1 Band structure

We will focus principally on the conduction band structure, since we are mainly interested in shifts in intra-band transitions and in the effect of the Fermi level's position, which we find to be relevant only if above the CBM. All systems considered are characterised by similar features in their band structure (see Fig. 1): the lowest miniband M1 is well separated from the upper minibands, and exhibits a minimum at the *Γ* point (*i.e.*, for *q*_*x*_ and *q*_*y*_ = 0) and maxima close to (or exactly at) the Brillouin zone boundary corners. The next set includes three minibands (M2, M3, M4), reflecting the p-like symmetry of the isolated quantum dot wave functions from which they derive (in case A this set includes higher minibands as well), followed by a continuum at higher energies (we consider the lowermost 7 minibands in systems A, C and D, and the lowermost 8 minibands in B, due to an additional degeneracy found in the band structure for that system). They all have minima close to (or exactly at) the Brillouin zone boundary corners, and maxima at the *Γ* point, *i.e.*, the exact opposite to M1. As a consequence the minimum separation between M1 and M2 is located at the zone boundaries, whereas the maximum energy separation, which can be much larger than for an isolated NC (see Table 1), occurs at the *Γ* point.

**Table 1:** Energy difference 
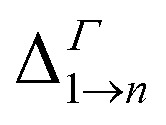
 (for *n* = 2, …, 5) at the *Γ* point in meV (as all Δ are calculated at *Γ*, we omit the superscript)

	Film				Isolated dot			
	A	B	C	D	A	B	C	D
Δ_1→2_	930	779	545	483	648	706	470	475
Δ_1→3_	969	838	576	558	669	726	502	506
Δ_1→4_	1019	843	577	569	669	726	502	506
Δ_1→5_	1060	961	892	828	815	880	815	814

System A exhibits a larger miniband intermixing, owing to its small size and the specific nature (anion-rich) of its surface. Interestingly system B, which is obtained from system A by exchanging anions and cations, exhibits a dramatically different miniband structure because of the significantly reduced wave function overlap between neighbouring dots originating from the different type of surface atoms. Such overlap is similarly small in systems C and D, due to their large size,^3^ leading to flatter minibands (M4 in particular). In all systems, with the only exception of system C, M2 features a flat region close to the *Γ* point, especially pronounced in system D.

**Fig. 2:**
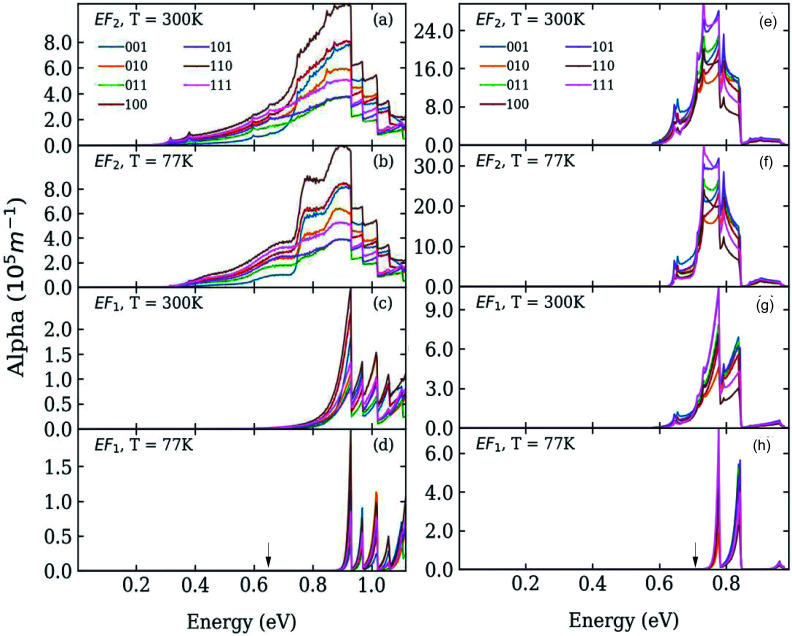
Absorption coefficient in systems A (left) and B (right) where the dots are separated by one bond length, at *T* = 77 K (panels b, d, f and h) and *T* = 300 K (panels a, c, e and g), calculated for different directions of the light polarization and for different values of the Fermi energy *E*_F_: at the bottom (panels c, d, g, and h), and in the middle (panels a, b, e, and f) of the lowest miniband (M1). The calculated position of the lowest absorption peak in isolated dots is indicated by black arrows in the bottom panels (d and h).

**Fig. 3:**
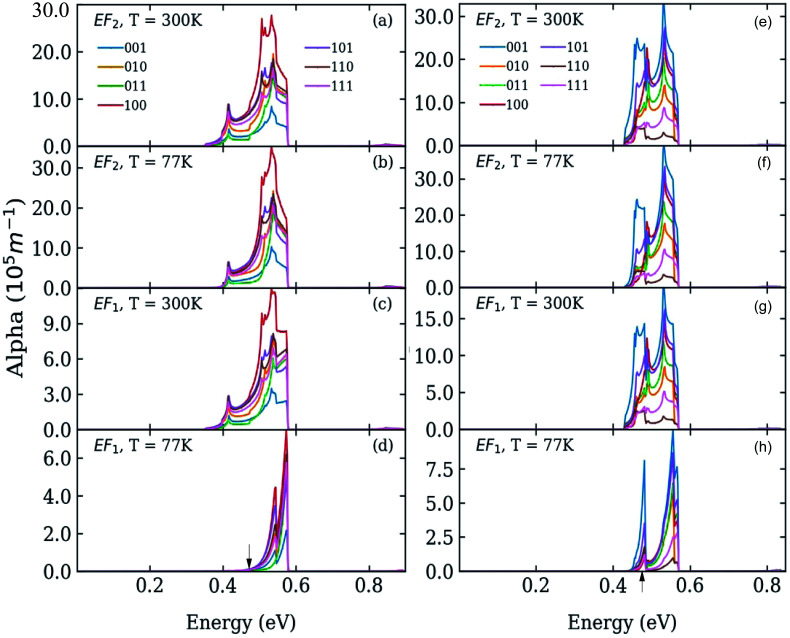
Absorption coefficient in systems C (left) and D (right) where C-type and D-type dots are separated by 0.25 and 1 bond lengths, respectively, at *T* = 77 K (panels b, d, f and h) and *T* = 300 K (panels a, c, e and g), calculated for different directions of the light polarization and for different values of the Fermi energy *E*_F_: at the bottom (panels c, d, g, and h), and in the middle (panels a, b, e, and f) of the lowest miniband (M1). The calculated position of the lowest absorption peak in isolated dots is indicated by black arrows in the bottom panels (d and h).

Due to its importance in absorption, we report in Table 1 the energy difference 
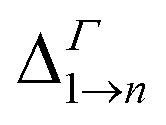
 between bands M1 and Mn at the *Γ* point, and compare it with the energy separation between the same levels in isolated dots, where it corresponds to the position of the lowermost inter-band absorption peak (black arrows in Fig. 2, 3, S1, and S2 ESI†).

We find that all values of 
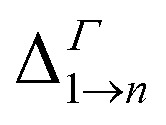
 in the films are *blue-shifted* compared with the same energy separations in isolated dots. The shifts range from about 8 meV, for 
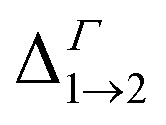
 in system D, to as much as 350 meV for 
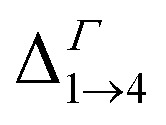
 in system A. As a consequence, if an intra-miniband transition in the film originates from the *Γ* point, we expect its energy to be blue-shifted: in the case of the (intra-miniband) absorption edge, Table 1 would predict blue shifts of up to 282 meV (system A).

### 3.2 Absorption coefficient

We calculated the absorption coefficient for different inter-dot separations, temperatures, Fermi level positions and light polarizations. Fig. 2 and 3 show the results for systems A–D, when A, B and D quantum dots are one bond length apart, whereas C dots are 0.25 bond lengths apart (
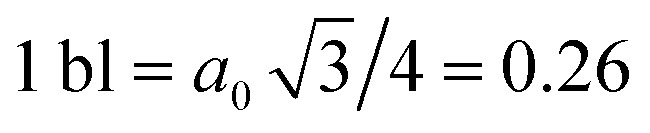
 nm for InAs, where *a*_0_ is the bulk lattice constant) – the closest separation considered here – at two different temperatures (77 K and 300 K) and for two different positions for the Fermi level: at the bottom (*E*_F1_) and in the middle (*E*_F2_) of M1, (*e.g.*, *E*_F1_ = −4.697 eV and *E*_F2_ = −4.503 eV – relative to the vacuum energy - respectively, for system A), corresponding to low and high doping levels (or a low/high carrier injection from neighbouring regions), respectively.

**Fig. 4:**
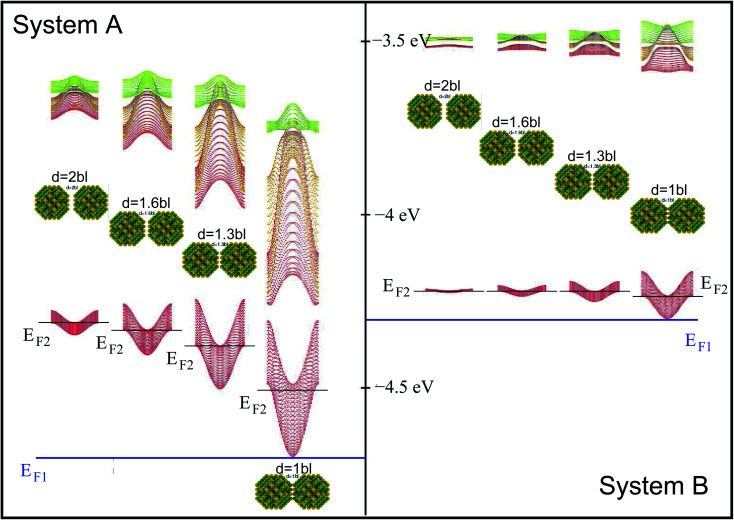
Miniband structure for the lowest conduction band states in structures A (left) and B (right) as a function of inter-dot separation (increasing from right to left – as shown by the distance between the nanocrystals), ranging from 1 to 2 bond lengths (bl). Also shown are the positions of *E*_F1_ (blue line) and *E*_F2_ (black lines) in all configurations. The energies are referred to the vacuum level.

**Fig. 5:**
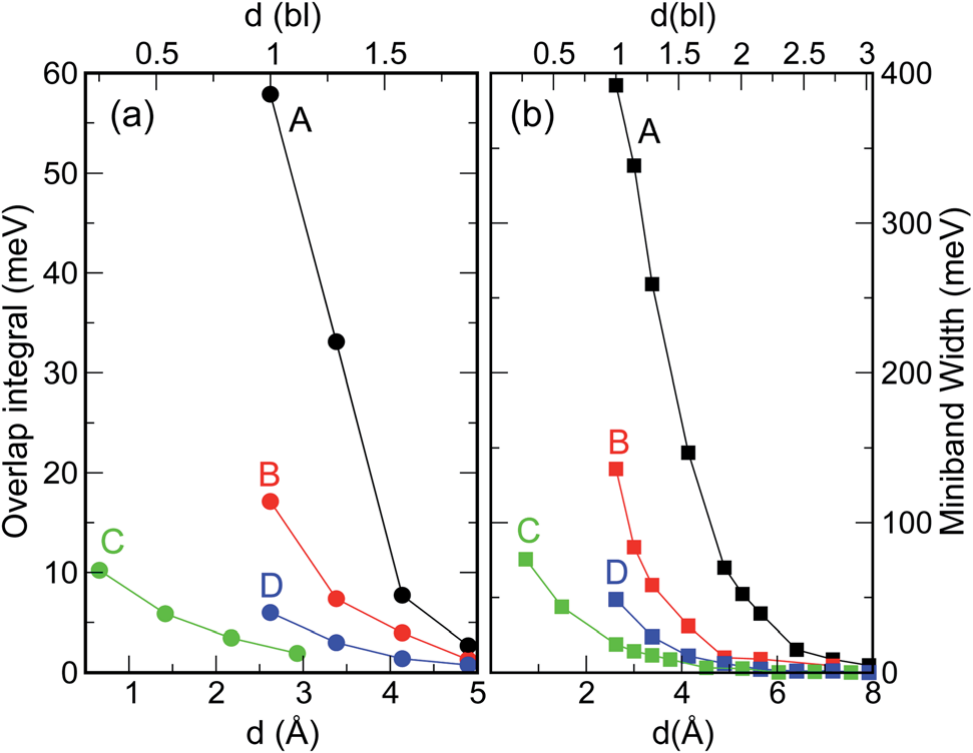
Effect of interdot separation on the film's band structure: (a) coupling energy (*i.e.*, overlap integral *V*_11_ = |〈*ψ*_1_(*r*)|*V*(*r*)|*ψ*_1_(*r* − *r*′)〉|; here *ψ*_1_(*r*) is the single dot wave function for state 1 – the CBM – and *V*(*r*) is the potential), and (b) miniband width, calculated for M1 as a function of separation (indicated in both Å, lower *x* axis, and bond lengths, upper *x* axis), for all structures considered here. These quantities were calculated for *d* = 1 bl in films made of different materials (InAs, InSb, CdSe, and PbSe) in ref. 3.

When considering larger inter-dot separations, we will keep the *absolute* position of *E*_F1_ and the *relative* position of *E*_F2_ fixed. This means that, as displayed in Fig. 4, *E*_F1_ will be found progressively deeper into the band gap, while *E*_F2_ will remain in the middle of M1, as the energy of the bottom of M1 increases (*i.e.*, moves upwards), due to the decrease in inter-dot coupling (Fig. 5(a)) – hence mininband width (Fig. 5(b)) – occurring with increasing separation. This will allow us to investigate the effects of both a constant and a varying Fermi level.

#### 3.2.1 Effect of light polarization

We find that, for each system, the positions of absorption thresholds and absorption peaks are rather independent of light polarization for each particular Fermi level and temperature, whereas their amplitude is more sensitive to it, although remaining approximately within the same order of magnitude for the different polarization directions. The order of magnitude of the absorption coefficient for all the systems considered is around 10^5^ to 10^6^ m^−1^, similar to what is predicted for InAs/GaAs epitaxial dot arrays,^15^ suggesting an efficient absorption.

Interestingly we find that the same light polarization affects each system in a different fashion, depending on the symmetry of the wave functions in the various cases: while 110-polarized light yields the strongest absorption in A, it has the opposite effect in B and D; similarly, polarization along 100 leads to the highest absorption C but not in B and D, where the absorption is strongest for light polarized along 111 and 001, respectively.

#### 3.2.2 Effect of *T*

For *E*_F_ = *E*_F1_ and *T* = 77 K, M1 is mainly occupied around the *Γ* point, *i.e.*, the lowest energy in the miniband: the calculated absorption peaks occur at the values of Δ_1→*m*_ (see Table 1), indicating that the absorption threshold is closely related to the 1 → 2 transitions taking place around *Γ*. At higher temperatures (*T* = 300 K) the absorption peaks remain at the same positions, but they are broadened to the red, due to the increased (thermal) occupation of higher energy states within M1 (which are closer to M2), leading to an enhanced photon absorption at lower energies.

In the case of systems B and C this broadening is also accompanied by the appearance of additional low-energy peaks leading to a substantial red shift in the absorption threshold. This is due to the small width of M1 in these systems (136 meV and 81 meV, respectively – see Fig. 5(b)) that leads to a more uniform occupation, and hence to a greater reduction of the absorption threshold energy due to the efficient photon absorption from M1 to M2 from every *q⃑* -state. If M1 is wide, however, only the states around the *Γ* point are occupied, and therefore the absorbed photons are mainly those with energies close to Δ_1→2_ (as in the case of systems A and D).

For *E*_F_ = *E*_F2_ the spectra exhibit little dependence on temperature. As the Fermi level is placed in the middle of M1 all the states around the *Γ* point are completely occupied, and so are also states higher in energy. The 1 → *n* transitions for different *q⃑* cover a wide range of energies, most of which are lower than Δ_1→*n*_. Therefore the absorption threshold energy is significantly reduced. In this case the flatter M1, the smaller the temperature-induced energy red shift of the absorption threshold, when compared to the case *E*_F_ = *E*_F1_.

#### 3.2.3 Effect of the position of the Fermi level *E*_F_

A change of the Fermi level position from *E*_F1_ (the bottom of M1) to *E*_F2_ (the middle of M1) affects the occupation of minibands, and therefore the absorbed photon energies, proportionally to the width of M1 (see Fig. 4). For *E*_F_ = *E*_F2_, system A exhibits, at both 77 K and 300 K, a considerable reduction in the absorption threshold position and a change in absorption coefficient behaviour as a function of energy, which shows a linear increase from very low energies, as opposed to the exponential increase found for *E*_F_ = *E*_F1_. This is due to the strong inter-dot coupling in this system (see Fig. 5(a)), yielding both wide minibands and large miniband intermixing (see Fig. 1), that leads to enhanced absorption for a wider range of photon energies.

The red shift of the intra-conduction-band absorption threshold, when moving from *E*_F1_ to *E*_F2_, is also predicted to occur, albeit to a lesser extent, in all the other systems, where it will, however, be more evident at low temperatures, due to the similar way in which temperature and Fermi level position affect the lower miniband's occupation.

#### 3.2.4 Effect of inter-dot separation

**Fig. 6:**
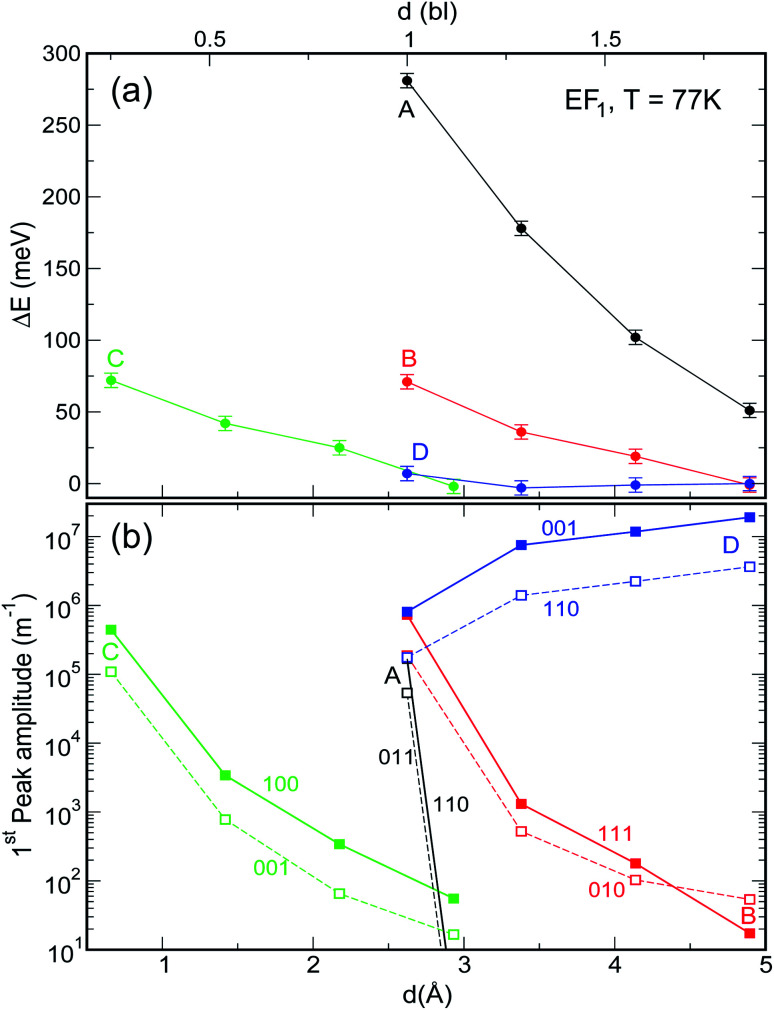
Effect of increasing interdot separation – expressed both in bond lengths (upper *x* axis) and Å (lower *x* axis) – on (a) the energy shift Δ*E* and (b) the amplitude, of the lowermost absorption peak in films of NCs of type A–D for *E*_F_ = *E*_F1_ and *T* = 77 K. (Full spectra are shown in panels d and h in Fig. S1 and S2 ESI†). Δ*E* is calculated with respect to the position of the first transition in isolated dots. In the case of system C, the dots are closer together due to the interlocking of the surface atoms.

**Fig. 7:**
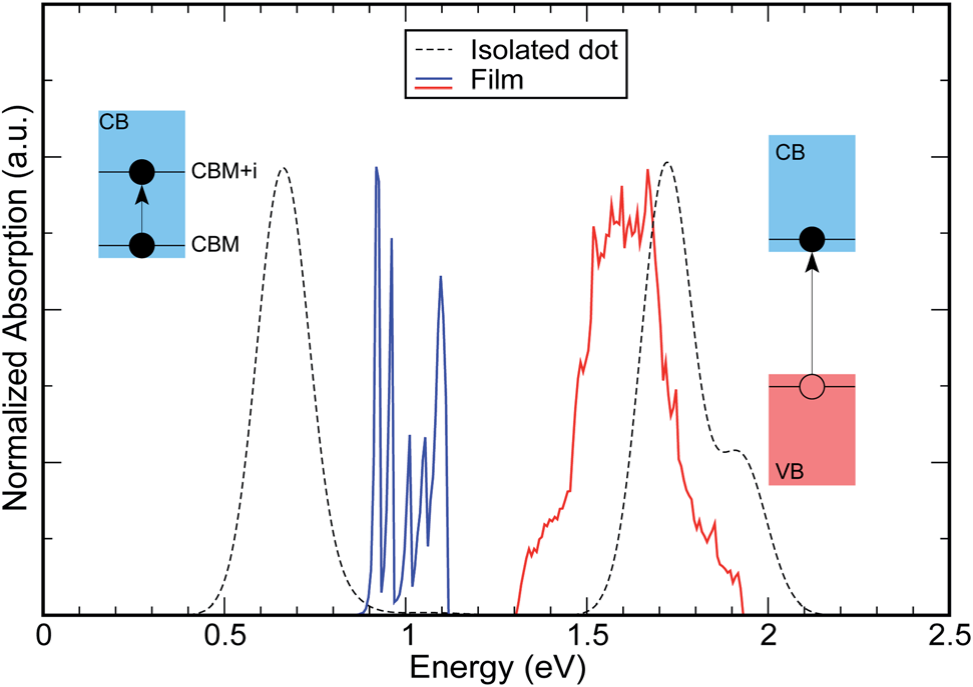
Comparison between the absorption spectra of (i) an isolated (dashed lines) InAs CQD with *R* = 1.2 nm (structure A) and (ii) a film (solid lines) made of these nanostructures (As we are mainly interested in the position of the peaks, the amplitude of some of them has been rescaled for better comparison). Although the band gap absorption is red shifted in the film (red solid line), the intra-band absorption is strongly blue shifted (blue solid line), compared to the isolated dot.

When the distance between the dots is increased, their coupling decreases leading to a flattening of all minibands^3^ (see Fig. 5 and 4), with the electronic structure tending to the discrete levels of the isolated dot, for large separations. The effect of this flattening is the more pronounced, the wider M1 for the lowest inter-dot separation: system A, therefore, is affected more dramatically than the other systems, as can be seen from Fig. 6(a) [the variation of the full spectra with increasing distance is displayed in Fig. S1 and S2 (ESI†)], which focuses on the case *E*_F_ = *E*_F1_ and *T* = 77 K (*i.e.*, panels d and h in Fig. S1 and S2 ESI†). The main effects of increasing interdot separation are: (i) the increasing red shifts of the intraband absorption edge – Fig. 6(a) – (and of all other peaks), and (ii) the decreasing magnitude of the absorption coefficient – Fig. 6(b). The former feature evidences the progressive *blue* shift in the intra-band absorption edge occurring when transitioning from the dilute solution limit (large inter-dot distance) to close packed arrays (minimum dot-to-dot separation), which starkly contrasts with the *red* shift of the band gap absorption that we predict (see Fig. 7), and that has been observed experimentally,^4,9–12^ in the same conditions.

As discussed above, both effects (i) and (ii) are more pronounced in system A, where the peaks can shift by more than 250 meV and the absorption coefficient decreases by as much as 22 orders of magnitude for inter-dot separations ranging from 1 to ∼2 bond lengths, in contrast to a maximum variation of about 4 orders of magnitude for systems B and C. This striking difference in absorption reduction between system A on one side, and systems B and C on the other, is due to the fact that, considering the larger width of M1 (*i.e.*, stronger coupling) in the former system, when *E*_F_ = *E*_F1_, the distance from M1 to *E*_F_ increases more rapidly with increasing dot-to-dot separation (*i.e.*, decreasing coupling) for A than it does for the other systems, in which the band is already much flatter and the coupling much weaker for the closest inter-dot separation (see Fig. 5 and 4). In other words, given that the width of M1 in system A is 392 meV and *E*_F_ is at its bottom for the shortest inter-dot separation, when this separation is increased the band's bottom can shift upwards by 392 meV increasing its distance from *E*_F_ by as much, whereas a narrow band such as M1 in D (width = 48 meV) can only move 48 meV away from *E*_F_. The reduction in absorption is proportional to the distance between M1 and *E*_F_.

The increase in temperature modifies the peak profiles, broadening the absorption threshold to the red, as commented above. At room temperature, the reduction in the magnitude of the absorption coefficient in system A is not as large as at low temperatures (the maximum reduction is only of about 5 orders of magnitude).

**Fig. 8:**
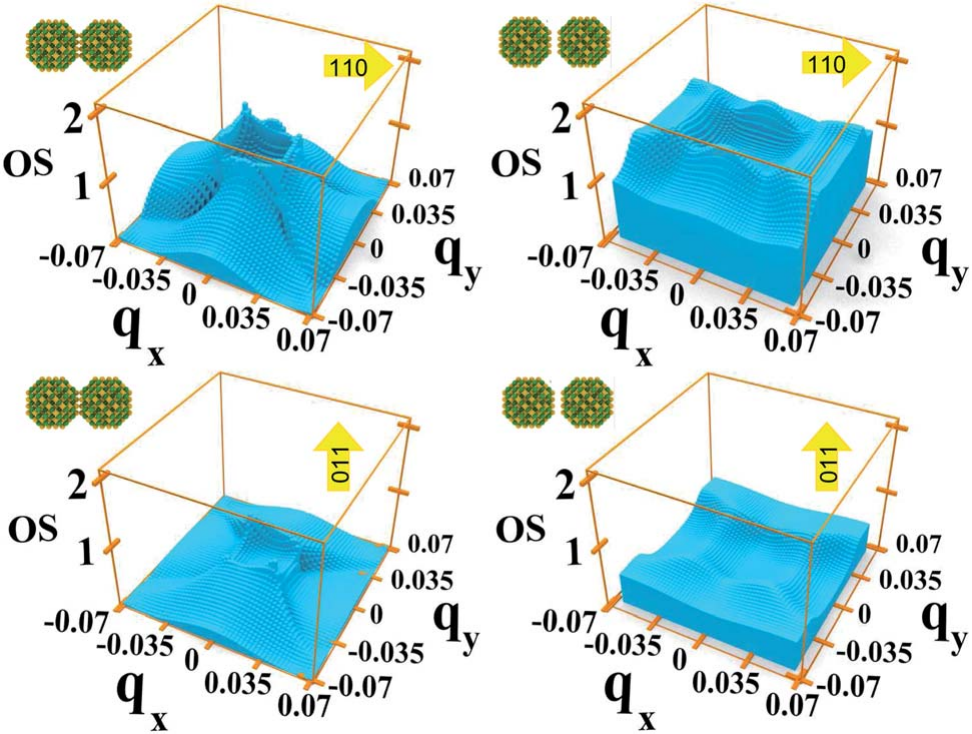
Oscillator strength relative to the 1 → 2 transition for each *q⃑* in system A with dots one bond length apart (left-hand panels), and two bond lengths apart (right-hand panels), calculated for two different directions of the light polarization: 110 (upper panels) and 011 (lower panels).

As discussed before, when *E*_F_ = *E*_F2_ the effects of temperature are negligible. The most remarkable feature we predict for this position of the Fermi level is the slight increase in absorption with inter-dot distance. The explanation for this effect is again related to the miniband flattening for greater inter-dot separations, and the interplay between miniband occupation and oscillator strength far from *Γ*. Indeed, we find that the main contribution to the variations in the absorption properties of the film with separation comes from the changes in the occupation of the different minibands, *f*(*E*_n_) in eqn (1). The magnitude of the oscillator strength2
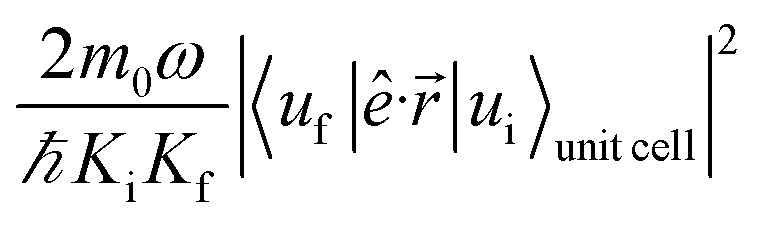
for the M1 → M2 transition, although nearly unchanged at *Γ*, increases strongly with inter-dot separation at the Brillouin zone boundaries (this is particularly evident in Fig. 8 for 110 polarization) leading to stronger absorption when M1 is flatter, hence more uniformly populated for all values of *q⃑*. This is exactly what happens for *E*_F_ = *E*_F2_, when the flattening of the minibands also leads to a narrowing of the range of absorption energies (as the M1–M2 separation is more uniform), compared to systems with wider minibands. As a consequence, the absorption peaks become not only higher but also narrower for greater inter-dot distances. This is also the origin of the increase, with increasing separation, in the first absorption peak amplitude predicted for system D in Fig. 6(b).

### 3.3 Oscillator strength

Our results show that both the position and the magnitude of the absorption threshold for *E*_F_ ≤ *E*_F1_ are determined by the transition from the minimum of M1 to the maximum of M2, both of which are located at the *Γ* point.

Interestingly, although the absorption threshold is of the order of hundreds of meV, the minimum gap between these two minibands (generally located at the Brillouin zone boundaries) could be as small as 50 meV (see Fig. 1).

As we discussed previously, one of the reasons for the greater absorption around the *Γ* point is the electron population of the minimum of M1. However, we find that transitions from the Brillouin zone center also have a larger oscillator strength eqn (2) than transitions from any other region in *q⃑* -space.

In system A the highest (lowest) absorption values are, in general, observed for light polarization along the 110 (011) direction. Fig. 8 shows the oscillator strength in the whole Brillouin zone for transitions from M1 to M2 for these light polarizations and for two different values of interdot separation: As expected, the 110 direction shows higher values than 011, by about a factor of 4, reflecting the difference found in the absorption curves. More importantly, for the closest dot-to-dot separation, the oscillator strength is orders of magnitude larger around *Γ* than at the Brillouin zone boundaries, demonstrating that the importance of the *Γ* point in absorption is not only related to the electron population, but also to the larger oscillator strength of transitions originating around that point.

**Fig. 9:**
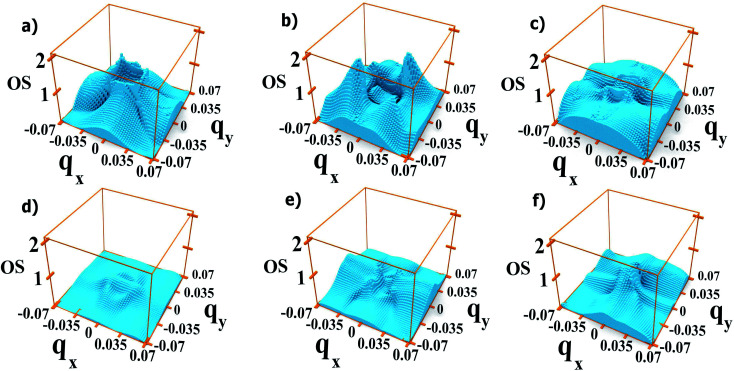
Oscillator strength for light polarized along the 110 direction, calculated for each *q⃑* in system A with dots 1 bond length apart, for transitions 1 → 2 (a), 1 → 3 (b), 1 → 4 (c), 1 → 5 (d), 1 → 6 (e), and 1 → 7 (f).

We find that the oscillator strengths for all 1 → *n* transitions with *n* = 2, …, 4 have a similar magnitude. The transitions from M5 upwards are much weaker, hence the reduction in amplitude of the absorption peaks at high energy. A similar behaviour is found for all polarizations. This effect is illustrated in Fig. 9 where we show the oscillator strength for transitions 1 → *n* (2 ≤ *n* ≤ 7) in system A. Here the strongest transitions are found between M1 and the miniband triplet M2, M3, M4. Higher energy minibands are split from this set, and the oscillator strength is much reduced. Indeed we find that the contribution of the M1 → Mn transitions with 4 < *n* ≤ 7 to the absorption coefficient in Fig. 3(a) is negligible.

## 4 Discussion of available experimental data

Intraband absorption in single InAs NCs was observed experimentally by Krapf *et al.*^16^ (and the relative transition energies calculated theoretically with different approaches^17–19^), over a decade ago. However, although the first intraband absorption spectra had been measured in CdSe NCs nearly a decade before that,^20^ the use of CQDs as intraband photodetectors in the infrared was only implemented much later.^21^ Indeed, for this application to be exploited, the dot must be doped with two electrons in order to saturate the occupancy of the 1S level.^21,22^ Efficient and accurate self-doping was recently developed only for mercury chalcogenide HgX CQDs (with X = S, Se, Te), in films of which intra band absorption between 1S and 1P levels and between 1P and 1D levels was reported.^23^ The size of such HgX NCs is typically of the order of 1.7–3.5 nm (ref. 21 and 22) and their separation in the film can easily exceed 0.5–1.0 nm.^23^ Unfortunately, both of these characteristics prevent these systems from exhibiting appreciable blue shifts in the film intraband transition energies, since, as we have explained above, the interdot coupling responsible for such shifts decreases crucially with (a) interdot distance and (b) dot size (Fig. 5). Furthermore, should even small shifts occur, the present experimental accuracy would be insufficient (with typical absorption peak full width at half maximum values of about 120 meV (ref. 24)) to detect them. All these factors contribute to make the intraband spectra of HgX dot arrays indistinguishable from those of single dots at present. These conclusions are confirmed by the excellent agreement found between the experimentally measured positions of the intraband absorption peaks in *films* of HgS NCs and the predictions of 2-band *k*⋅*p* calculations for *isolated dots*.^24^

Another important aspect worth discussing is the inter-dot separation realistically achievable in technologically relevant CQD films. The dot proximity (hence their coupling) in such arrays is limited by the presence of the capping ligands, routinely employed to stabilize the surface of these nanostructures, whose length may vary from ∼2 nm for oleic acid,^25^ to 0.35 nm for oxalic acid.^6^ A further reduction of the dot-to-dot distance down to 0.1 nm can be achieved by the use of inorganic ligands, such as atomic halide anions (Cl^−^, Br^−^ and I^−^),^26,27^ which can lead to the observation of band-like transport, high electron mobility and high photoconductivity in CQD films.^28^ Reactive self-assembly^29–31^ or laser annealing^32^ can finally lead to a facet-to-facet bonding of the dots. The range 1 ≤ *d* < 2 bl (0.26 ≤ *d* < 0.5 nm) considered in this work represents therefore a realistic estimate of the inter-dot separations achievable in experimental films.

## 5 Application to IBSCs

We have shown that strong inter-dot coupling leads to the formation of wide conduction minibands and that the presence of such wide minibands translates into (a) a *red* shift of the VBM → CBM band gap absorption transition, consistent with what is observed experimentally in many different materials^4,9–12^ when dilute solutions are replaced by close-packed solids and (b) a *blue* shift in the CBM1 → CBM2 inter-band absorption edge, which can be of the order of several hundreds of meV, depending on the dot size, (see Fig. 7), and which, to our knowledge, has never been reported before. Both shifts decrease with increasing inter-dot distance, until they converge, respectively, to the band gap absorption edge (a) and the *e*_1_ − *e*_2_ energy level separation (b) of the isolated dots, for sufficiently large distances.

We have also previously shown^34^ that allowed optical transitions exist between VB minibands and M1, and between M1 and higher energy conduction minibands Mn, with *n* ≥ 2. M1 can therefore be considered as a sort of “stepping stone”, or intermediate band (IB), for transitions from the VB minibands to CB minibands Mn with *n* > 1. This mechanism is similar to that at the base of the intermediate band solar cell (IBSC) concept. The difference is that, instead of exploiting sub-bandgap photon absorption, this scheme would enable the harnessing of some of the energy in excess of the NC bandgap, but lower than the threshold for carrier multiplication,^33^ which would otherwise be lost to heating of the cell, with consequent degradation of its performance. We have recently investigated its viability,^34^ and the predicted maximum achievable solar energy conversion efficiencies were far in excess of the Shockley–Queisser^35^ limit.

## 6 Conclusions

We presented a detailed investigation into the optical properties of 2D InAs NC arrays of different sizes, stoichiometries and surface topologies. We studied the effect of temperature, interdot separation, light polarization and Fermi level position. Surprisingly we found that large blue shifts of up to hundreds of meV in the intra-band absorption edges for optical transitions within the conduction band, and red shifts in the band gap transition edge are both the manifestation of wave function delocalization over more than one dot, that accompanies strong NC coupling in the film. This counter-intuitive result is shown to originate from the interplay of (i) the opposite curvature of the s-derived and p-derived conduction minibands in *q*-space, (ii) the miniband occupation profile, and (iii) the very different magnitude of the oscillator strength calculated for transitions at *Γ* and elsewhere in the Brillouin zone. This result is unaffected by the light polarization direction, but temperature, position of the Fermi level and inter-dot separation play increasingly important roles in the determination of the magnitude of both shifts. As a consequence, it should be possible to tune the intra-conduction-miniband absorption energies in a NC film within a wide window by engineering the interdot distance, which can be easily accomplished by selecting suitably sized ligands.

## Conflicts of interest

In accordance with our policy on conflicts of interest. There are no conflicts to declare.

## Supplementary Material

NA-002-C9NA00647H-s001
