# Supplementary material for: Optical properties of nanocrystal films: blue shifted transitions as signature of strong coupling
Source: Nanoscale Adv. 2019 Nov 25;2(1):384–93. doi: 10.1039/c9na00647h (PMC9419254; doi:10.1039/c9na00647h)
Supplement: NA-002-C9NA00647H-s001 [file NA-002-C9NA00647H-s001.pdf]

Electronic Supplementary Material (ESI) for Nanoscale Advances.
This journal is © The Royal Society of Chemistry 2019

Supplementary Information for:

Optical properties of nanocrystal films: blue shifted transitions as signature of strong coupling

Erik S. Skibinsky-Gitlin,[†] Salvador Rodríguez-Bolívar,[†,‡] Marco Califano,[*,¶] and Francisco M. Gómez-Campos[†,‡]

Departamento de Electrónica y Tecnología de Computadores, Facultad de Ciencias, Universidad de Granada, 18071 Granada, Spain, CITIC-UGR, C/ Periodista Rafael Gómez Montero, n 2, Granada, Spain, and Pollard Institute, School of Electronic & Electrical Engineering, and Bragg Centre for Materials Research, University of Leeds, Leeds LS2 9JT, United Kingdom

E-mail: *m.califano@leeds.ac.uk

Theoretical method

The electronic states of the periodic system (Fig.1, main text) are obtained within the tight-binding approach, by expanding the superlattice wave functions in a basis of single quantum dot conduction band eigenstates $\phi_n(\vec{r})$ obtained within the atomistic semiempirical pseudopotential framework, as[1]

[*]To whom correspondence should be addressed
[†]Departamento de Electrónica y Tecnología de Computadores, Facultad de Ciencias, Universidad de Granada, 18071 Granada, Spain
[‡]CITIC-UGR
[¶]Pollard Institute, School of Electronic & Electrical Engineering, and Bragg Centre for Materials Research, University of Leeds, Leeds LS2 9JT, United Kingdom

$$\psi_{j,\vec{q}}\left(\vec{r}\right)=\sum_{m}\sum_{\vec{R}_n}b_{m,\vec{q},j}e^{i\vec{q}\cdot\vec{R}_n}\phi_m\left(\vec{r}-\vec{R}_n\right), \tag{1}$$

where $b_{m,\vec{q},j}$ are the coefficients of the expansion (that depend on the single quantum dot state m - we use up to 8 conduction band states of the isolated quantum dot -, the specific $\vec{q}$-vector of the superlattice reciprocal space, and the miniband index j), and $\vec{R}_n$ are the superlattice vectors. The wave functions Eq. (1) can also be expressed in terms of Bloch functions as

$$u_{n,\vec{q}}\left(\vec{r}\right)e^{i\vec{q}\cdot\vec{r}}=\left[\sum_{m}\sum_{\vec{R}_n}b_{m,\vec{q},j}e^{i\vec{q}\cdot\left(\vec{R}_n-\vec{r}\right)}\phi_m\left(\vec{r}-\vec{R}_n\right)\right]e^{i\vec{q}\cdot\vec{r}}. \tag{2}$$

The normalization constant can then be obtained from

$$1=\sum_{m}\sum_{l}\sum_{\vec{R}_n}\sum_{\vec{R}_p}b_{m,\vec{q},j}b^*_{l,\vec{q},j}e^{i\vec{q}\cdot\left(\vec{R}_n-\vec{R}_p\right)}\int_{\text{whole space}}\phi_m\left(\vec{r}-\vec{R}_n\right)\phi^*_l\left(\vec{r}-\vec{R}_p\right)d\vec{r} \tag{3}$$

Due to the translational symmetry of the system, the contribution to the summation of each $\vec{R}_p$ is identical, leading to

$$1=N_s\sum_{m}\sum_{l}\sum_{\vec{R}_n}b_{m,\vec{q},j}b^*_{l,\vec{q},j}e^{i\vec{q}\cdot\vec{R}_n}\int_{\text{whole space}}\phi_m\left(\vec{r}-\vec{R}_n\right)\phi^*_l\left(\vec{r}\right)d\vec{r}\equiv K_{\vec{q},j}N_s, \tag{4}$$

where N_s is the number of quantum dots in the superlattice. The normalization constant is therefore $1/\sqrt{K_{\vec{q},j}N_s}$.

The absorption coefficient is calculated within the electric dipole approximation using Fermi's Golden Rule, as

$$\alpha(\hbar\omega)=\frac{2\pi e^2}{Q_{st}\nu_{\text{unit cell}}n_r c\epsilon_0\Delta E}\sum_{f}\sum_{i}\frac{\omega}{K_iK_f}\left|\hat{e}\cdot\langle u_f|\vec{r}|u_i\rangle_{\text{unit cell}}\right|^2\left(f(E_i)-f(E_f)\right), \tag{5}$$

where e is the electron charge, Q_{st} is the number of vectors of the reciprocal space for

which the Schrödinger equation is solved (a 501×501 grid discretization is used here to sample the Brillouin zone), $\nu_{\text{unit cell}}$ is the volume of the superlattice unit cell, n_r is the refractive index of the material (for simplicity here we use $n_r = 1$), c is the speed of light in vacuum, ϵ_0 is the vacuum dielectric constant, ΔE is the interval width within which energy is assumed to be conserved, (i.e., the Dirac's delta function is approximated as a box) ω is the angular frequency of the photon involved in the absorption process, $\hat{e}$ is the potential vector polarization, u_f and u_i are the Bloch functions of the superlattice wave function for the final (f) and initial (i) states, and $f(E)$ is Fermi-Dirac's statistics.

Calculated absorption spectra for different inter-dot separations

Table S 1: Multiplication factors fn by which the different curves in Figure S 1 have been rescaled (f1 refers to the red curves, f2 to the cyan, f3 to the green, and f4 to the violet curves, respectively).

Figure S 1								
	a	b	c	d	e	f	g	h
f1	1	1	1	1	1	1	1	1
f2	1	1	10^3	10^{13}	1	1	1	10^2
f3	1	1	10^4	10^{19}	1	1	1	10^3
f4	1	1	10^5	10^{23}	1	1	1	10^3

Table S 2: Multiplication factors fn by which the different curves in Figure S 2 have been rescaled (f1 refers to the red curves, f2 to the cyan, f3 to the green, and f4 to the violet curves, respectively).

Figure S 2								
	a	b	c	d	e	f	g	h
f1	1	1	1	1	10	10	10	10
f2	1	1	1	10^2	1	1	1	1
f3	1	1	1	10^3	1	1	1	1
f4	1	1	1	10^3	1	1	1	1

References

(1) Gómez-Campos, F. M.; Rodríguez-Bolívar, S.; Califano, M. High-Mobility Toolkit for Quantum Dot Films *ACS Photon.* **2016**, *3*, 2059-2067.

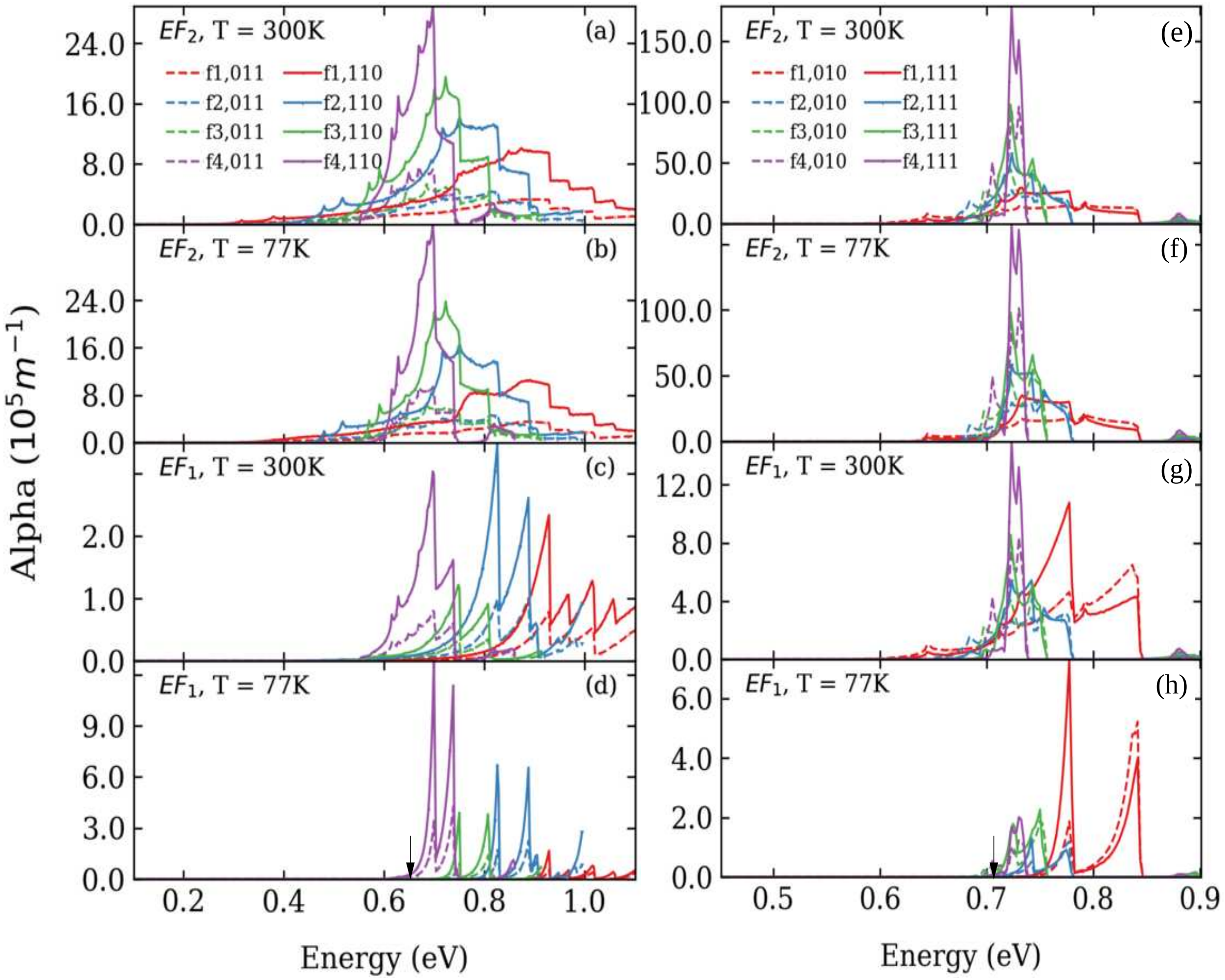

Figure S 1: Absorption coefficient in systems A (left) and B (right) calculated for different inter-dot separations (from one bond length, red lines, to 2 bond lengths, violet lines), at $T = 77$ K (panels b, d, f and h) and $T = 300$ K (panels a, c, e and g), calculated for different directions of the light polarization and for different values of the Fermi energy E_F: at the bottom (panels c, d, g, and h), and in the middle (panels a, b, e, and f) of the lowest miniband (M1). For convenience and for the sake of clarity, the curves in each panel have been multiplied by different factors fn (where f1 refers to the red curves, f2 to the cyan, f3 to the green, and f4 to the violet curves, respectively): their values are reported in Table Table S 1. The calculated position of the lowest absorption peak in isolated dots is indicated by black arrows in the bottom panels (d and h).

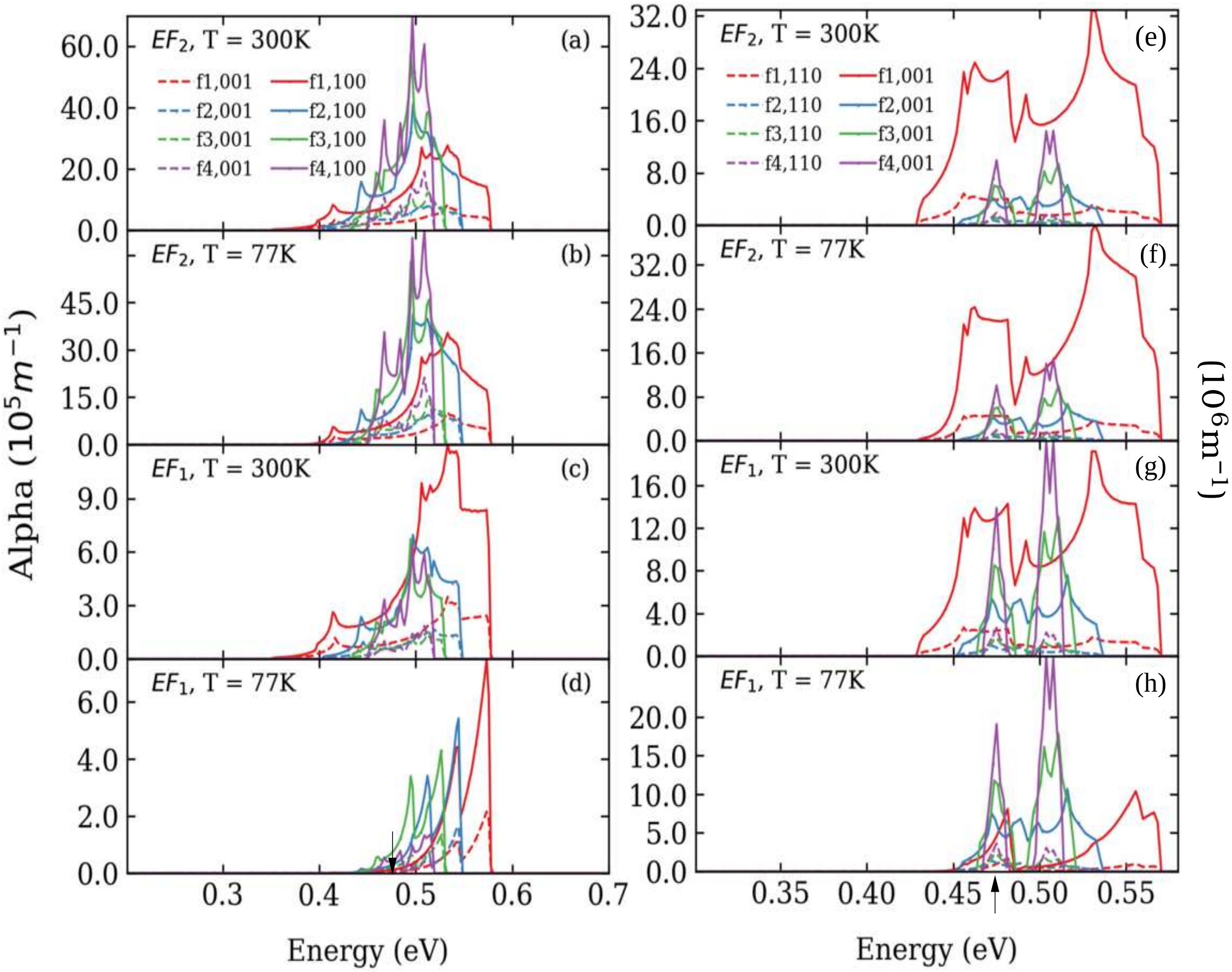

Figure S 2: Absorption coefficient in systems C (left) and D (right) calculated for different inter-dot separations (D: from one bond length, red lines, to 2 bond lengths, violet lines; C from 0.25 bond lengths, red lines, to 1.25 bond lengths, violet lines), at $T = 77$ K (panels b, d, f and h) and $T = 300$ K (panels a, c, e and g), calculated for different directions of the light polarization and for different values of the Fermi energy E_F: at the bottom (panels c, d, g, and h), and in the middle (panels a, b, e, and f) of the lowest miniband (M1). For convenience and for the sake of clarity, the curves in each panel have been multiplied by different factors fn (where f1 refers to the red curves, f2 to the cyan, f3 to the green, and f4 to the violet curves, respectively): their values are reported in Table Table S 2. The calculated position of the lowest absorption peak in isolated dots is indicated by black arrows in the bottom panels (d and h).
